# Association of Participation in a Value-Based Insurance Design Program With Health Care Spending and Utilization

**DOI:** 10.1001/jamanetworkopen.2023.2666

**Published:** 2023-03-13

**Authors:** Hui Zhang, David W. Cowling

**Affiliations:** 1 Now with Medicare and Duals Analytic Unit, California Department of Health Care Services, Sacramento; 2 Health Innovation and Pilot Performance Section, California Public Employees’ Retirement System, Sacramento

## Abstract

**Importance:**

Value-based insurance design (VBID) has mostly been used in improving medication use and adherence for certain conditions or patients, but its outcomes remain uncertain when applied to other services and to all health plan enrollees.

**Objective:**

To determine the association of participation in a California Public Employees’ Retirement System (CalPERS) VBID program with its enrollees’ health care spending and utilization.

**Design, Setting, and Participants:**

A retrospective cohort study with difference-in-differences propensity-weighted 2-part regression models was performed in 2021 to 2022. A VBID cohort was compared with a non-VBID cohort both before and after VBID implementation in California in 2019 with 2 years’ follow-up. The study sample included CalPERS preferred provider organization continuous enrollees from 2017 through 2020. Data were analyzed from September 2021 to August 2022.

**Exposures:**

The key VBID interventions include (1) if selecting and using a primary care physician (PCP) for routine care, PCP office visit copayment is $10 (otherwise, PCP office visit copayment is $35 as for specialist visit); and (2) annual deductibles reduced by a half through completion of the following 5 activities: annual biometric screening, influenza vaccine, nonsmoking certification, second opinion for elective surgical procedures, and disease management participation.

**Main Outcomes and Measures:**

The primary outcome measures included annual per member total approved payments for multiple inpatient and outpatient services.

**Results:**

The 2 compared cohorts of 94 127 participants (48 770 were female [52%]; 47 390 were younger than 45 years old [50%]) had insignificant baseline differences after propensity-weighting adjustment. The VBID cohort had significantly lower probabilities of inpatient admissions (adjusted relative odds ratio [OR], 0.82; 95% CI, 0.71-0.95), and higher probabilities of receiving immunizations (adjusted relative OR, 1.07; 95% CI, 1.01-1.21) in 2019. Among those with positive payments, VBID was associated with higher mean total allowed amounts for PCP visits in 2019 and 2020 (adjusted relative payments ratio, 1.05; 95% CI, 1.02-1.08). There were no significant differences for inpatient and outpatient combined totals in 2019 and 2020.

**Conclusions and Relevance:**

The CalPERS VBID program achieved desired goals for some interventions with no added total costs in its first 2 years of operation. VBID may be used to promote valued services while containing costs for all enrollees.

## Key Points

**Question:**

Was participation in a California public payer’s value-based insurance design (VBID) program associated with desired changes in health care spending and utilization?

**Findings:**

This retrospective cohort study included 94 127 enrollees in commercial health plans. The VBID cohort was associated with significantly higher spending on or use of primary care physicians and immunizations, lower inpatient admissions and surgical procedures, and similar changes in overall spending compared with a non-VBID cohort in 2019 or 2020.

**Meaning:**

Participation in this VBID program was associated with positive targeted changes in outpatient and inpatient spending and utilization without increasing total costs.

## Teaser

This cohort study evaluates the association of participation in a value-based insurance design program in California with health care spending and utilization.

## Introduction

Value-based insurance design (VBID) is a demand-side strategy to increase health plan member use of high value care and reduce low value care usage, primarily through cost-sharing tiers to direct member choice of preferred health care services, medications, and clinicians.^1,2,3,4,5^ VBID encourages members to engage in healthy activities and manage chronic conditions while discouraging unnecessary or unwarranted health care choices to improve member health and reduce total health care costs in the long term.

VBID has been implemented as a cost-sharing model mostly for certain prescription drugs or treatments for specific diseases or chronic conditions or select patient populations. Most studies have found VBID was associated with desired changes in targeted utilization and medication adherence, but its association with clinical outcomes and health care spending remain uncertain.^6,7,8,9,10^ VBID studies have been criticized for lacking rigorous study designs and statistical methods to help draw valid causal relationship conclusions.^7,11,12,13^

We examined a VBID program that was launched by the California Public Employees’ Retirement System (CalPERS) in one of its preferred provider organization (PPO) commercial health plans in 2019. The VBID program was mainly targeted at enhancing primary care for its PPO health plan enrollees. The program’s objectives are to provide economic incentives for members to receive high-value coordinated care at the right place and time through a personal primary care physician (PCP); to increase member engagement in health care decisions and reward members for engaging in healthy activities; and to improve outcomes and lower costs and premiums in the long term.

PPO enrollees can choose any in-network physicians when seeking care, which allows freedom of choice of clinician but also increases the risk of fragmented care and higher costs. The CalPERS VBID PPO personal PCP model aims to provide its members a place where their health issues will first be treated by their PCPs, who will guide them through the health care system when referrals to specialists are needed. PCPs also facilitate dialogue between members and clinicians, fostering member participation in decision-making about their own health care, and providing opportunities for disease prevention and health promotion as well as early detection and treatment of conditions, while controlling or reducing costs in the long term.^14^

The primary VBID interventions include a $10 office visit copayment if a member selects and uses a personal PCP for routine care, including mental health and substance use physician visits. Otherwise, a $35 office visit copayment is required for a PCP visit, the same as a specialist visit. Additionally, annual deductibles are reduced from $1000 to $500 for individuals and from $2000 to $1000 for families through completion of the following 5 activities with a $100 credit each for individuals (credit doubled for families): annual biometric screening, annual influenza vaccine, nonsmoking certification, second opinion for elective surgical procedures, and disease management participation. Finally, there is a waiver of inpatient co-insurance (20%) for delivery for expectant mothers enrolled in a healthy mother’s program, which provides tools and resources needed for a healthy pregnancy and delivery.

The objective of this study is to evaluate the association of participation in the CalPERS VBID program with health care costs and utilization by its enrollees in 2019 and 2020. We expected a significant VBID association with some of the target services but were unsure about its association with short-term total cost savings.

## Methods

This cohort study was reviewed as minimal risk and waived for informed consent by the Committee for the Protection of Human Subjects, the institutional review board for the California Health and Human Services Agency. This report follows the Strengthening the Reporting of Observational Studies in Epidemiology (STROBE) reporting guideline.

The VBID interventions were implemented in one of CalPERS PPO health plans, which we label as VBID PPO. We selected another CalPERS PPO plan that did not implement the VBID interventions for comparison, which we call non-VBID PPO. The VBID PPO and non-VBID PPO plan had the same benefit design in terms of copayment, coinsurance, maximum out-of-pocket (OOP) limit, and pharmacy coverage before VBID implementation. The non-VBID PPO had a broader provider (clinicians and hospital systems, etc.) network and higher premium than the VBID PPO, whose premium was set even lower in 2019. After VBID implementation, the VBID PPO annual deductible and PCP visit copayments changed as described previously, but there were no differential changes in copayments for specialist and emergency department visits, coinsurance rate for inpatient hospital admission, annual OOP maximum, and prescription drug coverage and copayments (eTable 1 in Supplement 1).

The VIBD and non-VBID PPO plans are offered during the annual open enrollment period for CalPERS members to compare and choose. The VBID PPO plan provided a mobile health consumer app to increase member engagement through personalized communication and education about the VBID-incentivized activities. CalPERS members also have access to online materials about the VBID PPO incentives, in addition to member outreach and provider communication. The VBID PPO health plan reported that until the fourth quarter of 2019, less than 30% of members had attributed PCPs. For the 5 incentivized activities, the uptake rates for disease management participation and a second opinion for surgery reached more than 90%. More than half of VBID PPO members received an influenza vaccine, and up to 70% finished a biometric screening and nonsmoking certification. Only 17% of expectant mothers enrolled in a healthy mother’s program.

We applied a retrospective cohort study design and a difference-in-differences (DID) approach to compare the relative annual changes in cost and use before and after the 2019 VBID implementation between a cohort of VBID PPO continuous enrollees and a cohort of non-VBID PPO continuous enrollees from 2017 through 2020. The data sources included CalPERS commercial PPO California member enrollment and claims data.

The outcome measures are total allowed payments per member per year for inpatient and outpatient health services. Allowed payments are negotiated prices between health plans and providers, which are often much lower than list prices or charges by providers. The control variables include member age group, sex, region, family relationship and tier, employer type, retirement status, clinical risk score categorical group in 2017 and 2018, and year indicator.

### Statistical Analysis

A 2-part DID generalized linear model (GLM) was applied: the first logit GLM model predicts whether an enrollee would have any use or positive payments or not, and the second log-gamma GLM model predicts conditional positive payments. All regression models are adjusted for a VBID PPO indicator, its interaction with a year indicator (using 2018 as the reference year), and the control variables, with the average treatment effect on the treated (ATT) weighting and enrollee-cluster robust SEs. The exponentiated coefficients of the interaction terms from the VBID and year indicators are the multiplicative DID estimators of interest: relative odds ratio in the logit GLM model and relative payments ratio in the log-gamma GLM model.^15^ ATT weighting is based on propensity scores predicted from a logistic regression of the probability of selecting the VBID PPO cohort on the control variables. ATT weighting sets weights of the treatment group as 1s and weights of the control group as the products of propensity scores and inversed (1-propensity scores) to weight the control group equivalent to the treatment group.

Inpatient cesarean-section measure was analyzed for female participants aged between 15 and 55 years old. A sensitivity analysis was performed for female participants with any inpatient deliveries.

All statistical hypothesis tests were 2-sided tests with a priori significance level of .05 using STATA version 14.2 (StataCorp) and SAS Enterprise Guide version 8.3 (SAS Institute). Data were analyzed from September 2021 to August 2022.

## Results

The study population consisted of 94 127 CalPERS PPO health plan non-Medicare members; 50% (48 770) were younger than 45 years old and 52% (47 390) were female. The VBID PPO longitudinal cohort enrollees were significantly younger, more likely to be male, dependent, not single, employed by the state or public agencies, not retired, living in Northern California other than the Bay Area or Sacramento, or healthier in 2017 and 2018 when compared with the non-VBID PPO cohort. However, all significant differences became insignificant after the ATT weighting adjustment, which helped balance the control variables and improved the comparability of the VBID and non-VBID PPO cohorts (Table 1).

**Table 1.** California Public Employees’ Retirement System VBID Study Longitudinal Cohorts Summary Statistics in 2019

Characteristic	VBID [n = 24 498], No. (%)	Non-VBID (n = 69 629)			*P* value^a^
		No.	%		
			Before ATT weighting	After ATT weighting	
Age, y					
0-14	4885 (19.9)	9884	14.2	20.2	.26
15-24	3735 (15.3)	10 764	15.5	15.1	
25-34	2317 (9.5)	2761	4.0	10.1	
35-44	4521 (18.5)	8523	12.2	18.5	
45-54	4832 (19.7)	14 649	21.0	19.3	
55-64	3836 (15.7)	19 268	27.7	15.3	
≥65	372 (1.5)	3780	5.4	1.5	
Sex					
Female	12 404 (50.6)	36 366	52.2	50.5	.78
Male	12 094 (49.4)	33 263	47.8	49.5	
Relation					
Dependent	8646 (35.3)	20 958	30.1	35.4	.98
Spouse	4890 (20.0)	15 807	22.7	19.9	
Subscriber	10 962 (44.8)	32 864	47.2	44.7	
Tier					
2-Party	4284 (17.5)	15 915	22.9	17.1	.30
Family	16 355 (66.8)	41 279	59.3	66.7	
Single	3859 (15.8)	12 435	17.9	16.2	
Employer					
California State University	2200 (9.0)	11 224	16.1	9.1	.16
Public agency	7551 (30.8)	18 904	27.2	31.7	
School	2430 (9.9)	10 865	15.6	9.9	
State	12 317 (50.3)	28 636	41.1	49.3	
Member status					
Active	22 962 (93.7)	59 103	84.9	93.9	.57
Retired	1536 (6.3)	10 526	15.1	6.2	
Region					
Bay Area	2141 (8.7)	11 962	17.2	8.7	.49
Los Angeles area	3486 (14.2)	17 919	25.7	14.1	
Other Northern California	11 972 (48.9)	17 629	25.3	49.7	
Other Southern California	5643 (23.0)	17 300	24.9	22.7	
Sacramento area	1256 (5.1)	4819	6.9	4.9	
Risk score 2017^b^					
0-0.9	17 587 (71.8)	40 811	58.6	72.3	.80
1-1.9	3600 (14.7)	12 878	18.5	14.5	
2-3.9	2172 (8.9)	9422	13.5	8.7	
4-8.9	886 (3.6)	4846	7.0	3.6	
≥9	253 (1.0)	1672	2.4	1.0	
Risk score 2018					
0-0.9	17 171 (70.1)	39 794	57.2	70.7	.68
1-1.9	3655 (14.9)	12 892	18.5	14.7	
2-3.9	2314 (9.5)	9720	14.0	9.2	
4-8.9	1034 (4.2)	5325	7.7	4.1	
≥9	324 (1.3)	1898	2.7	1.3	

Abbreviations: ATT, average treatment effect on the treated; PPO, preferred provider organization; VBID, value-based insurance design.

^a^ *P* values were calculating using χ^2^ tests after ATT weighting. Before ATT weighting, χ^2^ test results for the % differences between VBID PPO and non-VBID PPO are all significant, all *P* < .0001.

^b^ The risk score is the Diagnostic Cost Group risk score based on member's age, sex, and diagnosis information, which ranged from close to 0 to up to 147 in baseline years. Higher risk score predicts higher health care utilization or costs.

**Figure 1.**
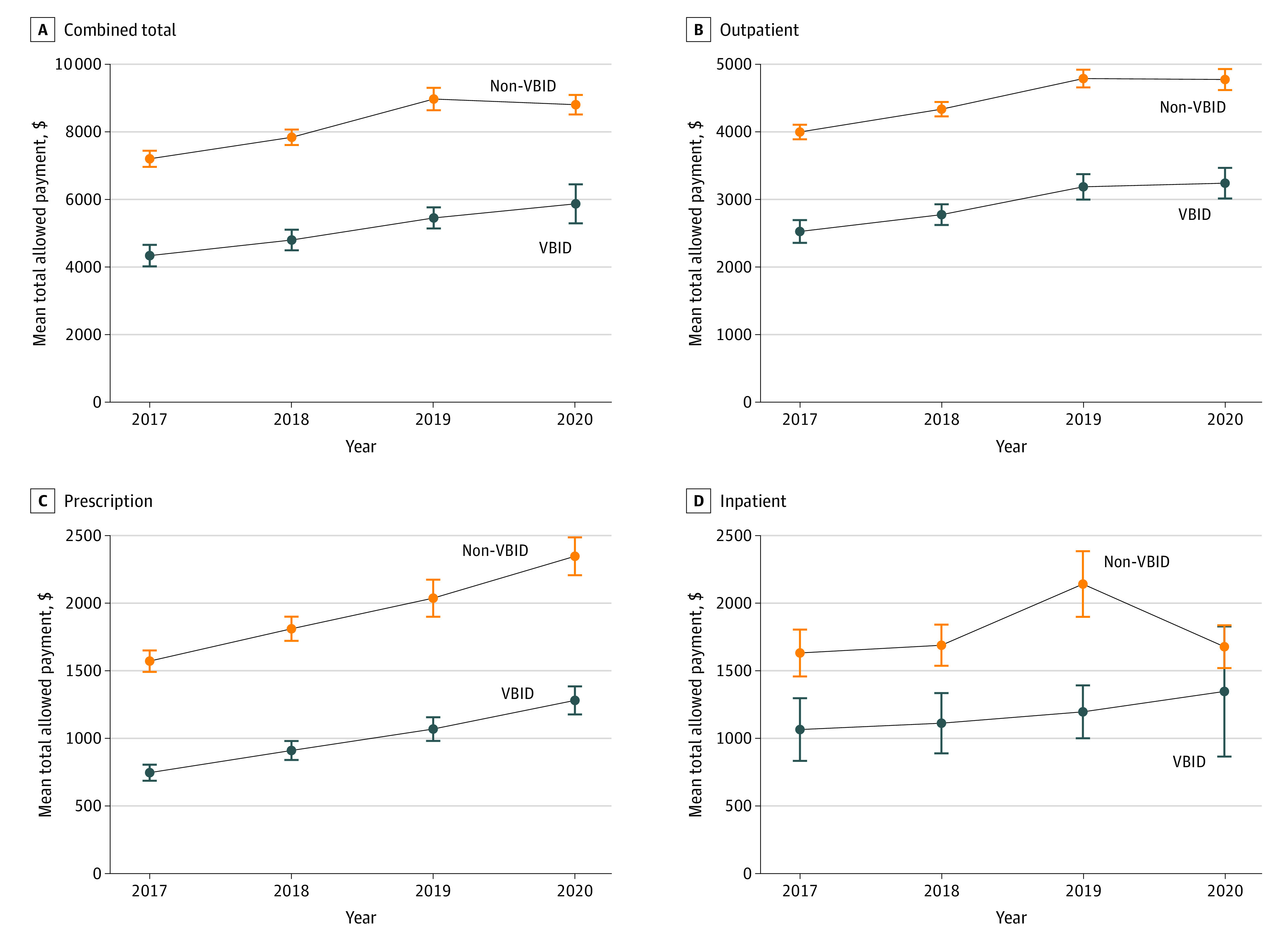
Unadjusted Yearly Trends of Selected per Member Annual Category Spending Measures by Cohorts Whiskers indicate 95% CI of mean. VBID indicates value-based insurance design.

**Figure 2.**
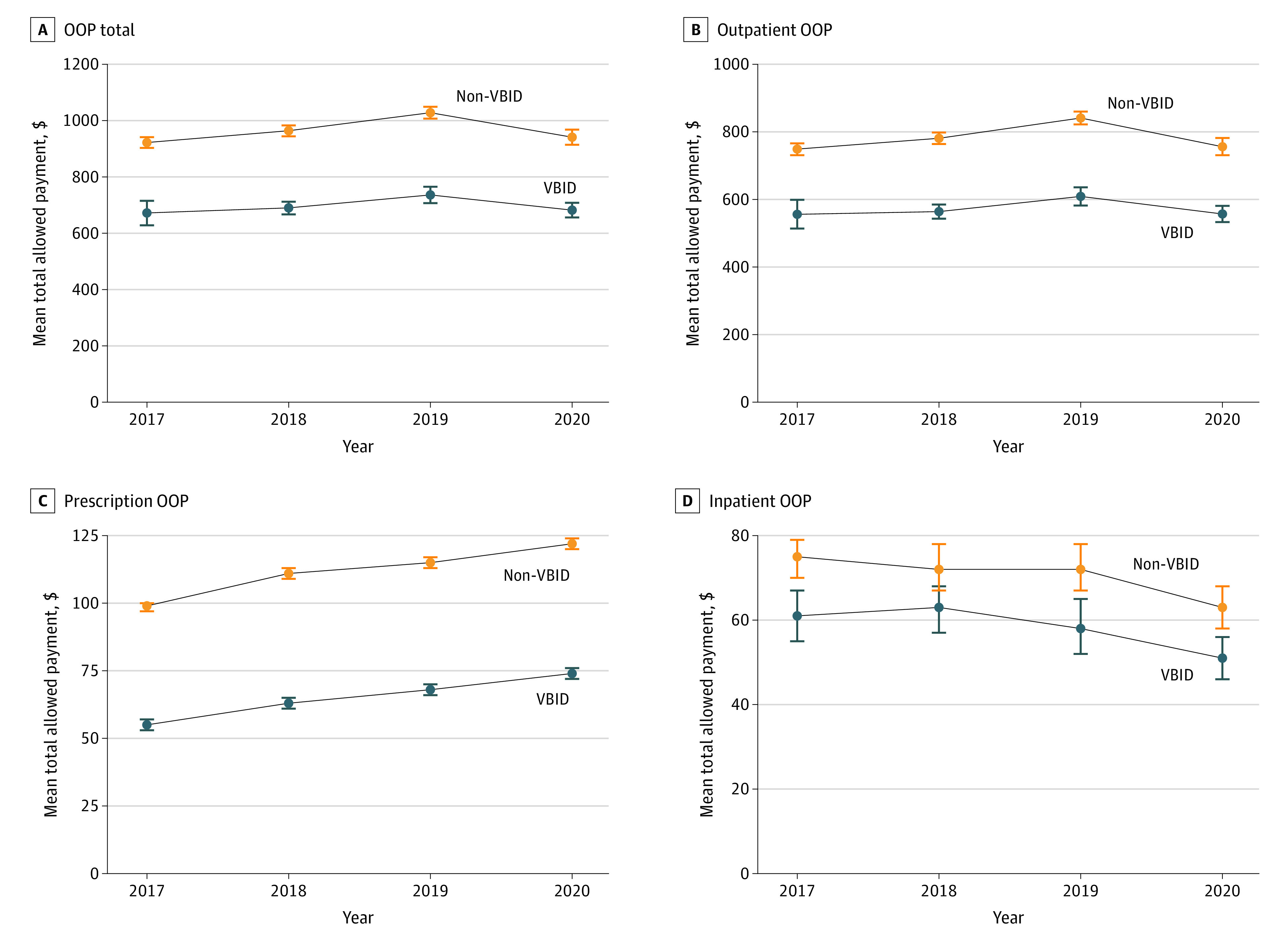
Unadjusted Yearly Trends of Selected per Member Annual Out-of-Pocket (OOP) Spending Measures by Cohorts Whiskers indicate 95% CI of mean. VBID indicates value-based insurance design.

**Figure 3.**
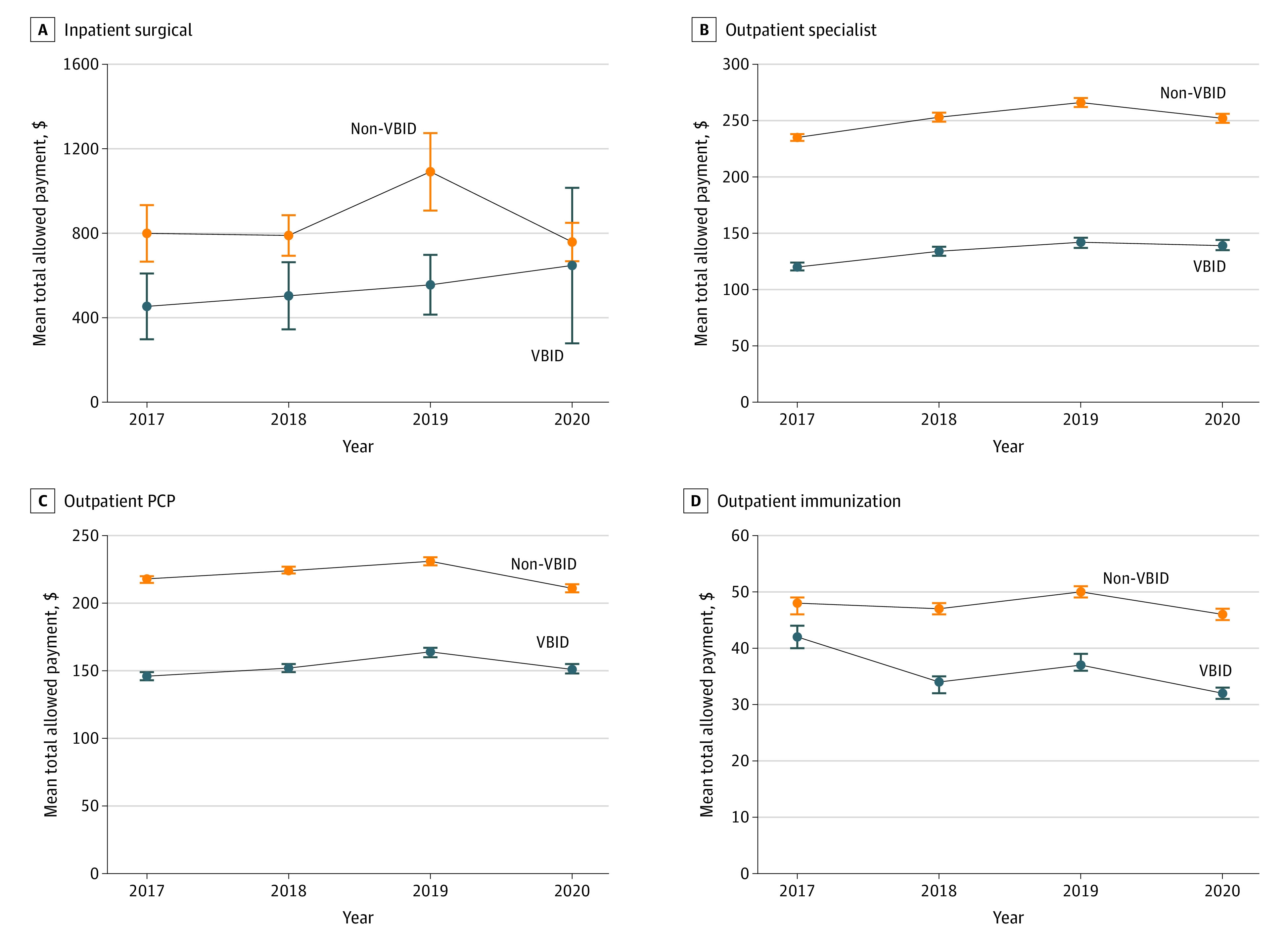
Unadjusted Yearly Trends of Selected per Member Annual Service Spending Measures by Cohorts Whiskers indicate 95% CI of mean. PCP indicates primary care physician; VBID, value-based insurance design.

The unadjusted per-member per-year mean total allowed payments for the VBID PPO were substantially lower than the non-VBID PPO, but with parallel pre-VBID trends. Many spending measures seem to be influenced substantially by the COVID-19 pandemic in 2020, which varied by service setting and type (Figures 1, 2, and 3).

The 2-part GLM regression-model-adjusted DID estimates are reported in Table 2. Compared with the non-VBID PPO cohort, the VBID PPO enrollees had significantly lower probability of inpatient hospital surgical admission in 2019 (adjusted relative odds ratio [OR], 0.74; 95% CI, 0.59-0.93; *P* = .01). The VBID PPO cohort also had a lower probability of total inpatient hospital admission and paying admission OOP in 2019 (adjusted relative OR, 0.82; 95% CI, 0.71-0.95; *P* = .008). The VBID PPO cohort had significantly higher chances of receiving immunizations in 2019 (adjusted relative OR, 1.07; 95% CI, 1.01-1.21; *P* = .01), seeing a PCP in 2020 (adjusted relative OR, 1.05; 95% CI, 1.01-1.10; *P* = .02), and paying for an outpatient visit OOP in 2020.

**Table 2.** Two-Part GLM Adjusted Difference-in-Differences Estimates 2019 to 2020^a^

Model and measure	2019			2020		
	Relative odds ratio (95% CI)	SE	*P* value	Relative odds ratio (95% CI)	SE	*P* value
**Logit GLM**						
Total allowed payment						
Total combined	0.99 (0.93-1.05)	0.03	.74	1.01 (0.95-1.07)	0.03	.86
Inpatient admission	0.82 (0.71-0.95)	0.06	.007	0.89 (0.77-1.03)	0.07	.12
Outpatient visit	1.00 (0.95-1.06)	0.03	.94	1.03 (0.98-1.09)	0.03	.23
Prescription drug	0.97 (0.93-1.01)	0.02	.13	0.99 (0.95-1.03)	0.02	.63
OOP						
Combined	1.01 (0.95-1.06)	0.03	.83	1.05 (0.99-1.11)	0.03	.08
Admission OOP	0.82 (0.71-0.95)	0.06	.008	0.88 (0.76-1.02)	0.07	.09
Visit OOP	1.01 (0.96-1.06)	0.03	.66	1.07 (1.01-1.12)	0.03	.01
Prescription OOP	0.97 (0.93-1.01)	0.02	.14	1.03 (0.98-1.07)	0.02	.25
Inpatient						
Inpatient cesarean section	0.77 (0.45-1.32)	0.21	.34	0.78 (0.46-1.34)	0.21	.37
Inpatient surgical	0.74 (0.59-0.93)	0.09	.01	0.85 (0.67-1.08)	0.1	.19
Outpatient						
PCP office visit	0.99 (0.95-1.03)	0.02	.69	1.05 (1.01-1.10)	0.02	.02
Specialist office visit	0.97 (0.93-1.01)	0.02	.20	1.00 (0.96-1.04)	0.02	.95
Psychiatrist outpatient	1.00 (0.94-1.06)	0.03	.97	0.99 (0.93-1.06)	0.03	.82
Emergency department	1.07 (1.00-1.15)	0.04	.06	1.01 (0.94-1.09)	0.04	.73
Laboratory test	0.97 (0.93-1.01)	0.02	.12	1.02 (0.97-1.06)	0.02	.50
Immunization	1.07 (1.01-1.12)	0.03	.01	1.03 (0.98-1.08)	0.03	.28
**Log-Gamma GLM**	**Relative payments ratio (95% CI)**	**SE**	***P* value**	**Relative payments ratio (95% CI)**	**SE**	***P* value**
Total allowed payment						
Total combined	1.03 (0.95-1.13)	0.05	.46	1.12 (0.96-1.31)	0.09	.15
Inpatient admission	0.91 (0.74-1.12)	0.1	.37	1.02 (0.77-1.34)	0.14	.89
Outpatient visit	1.05 (0.98-1.12)	0.04	.17	1.03 (0.95-1.13)	0.05	.45
Prescription drug	1.08 (1.00-1.17)	0.04	.06	1.10 (1.00-1.23)	0.06	.06
OOP						
Combined	0.98 (0.94-1.01)	0.02	.22	0.98 (0.93-1.03)	0.02	.37
Admission OOP	0.97 (0.87-1.08)	0.05	.59	0.90 (0.81-1.00)	0.05	.05
Visit OOP	0.98 (0.94-1.02)	0.02	.29	0.98 (0.93-1.03)	0.03	.50
Prescription OOP	1.02 (0.99-1.05)	0.02	.12	1.04 (1.00-1.08)	0.02	.03
Inpatient						
Inpatient cesarean section	1.29 (1.01-1.66)	0.16	.04	0.99 (0.77-1.27)	0.13	.92
Inpatient surgical	0.90 (0.66-1.24)	0.15	.54	1.17 (0.77-1.77)	0.25	.47
Outpatient						
PCP office visit	1.02 (1.00-1.05)	0.01	.04	1.05 (1.02-1.08)	0.01	.000
Specialist office visit	1.01 (0.97-1.04)	0.02	.71	1.01 (0.97-1.05)	0.02	.56
Psychiatrist outpatient	1.03 (0.95-1.11)	0.04	.53	1.02 (0.93-1.11)	0.05	.67
Emergency department	1.02 (0.93-1.12)	0.05	.72	1.03 (0.94-1.13)	0.05	.47
Laboratory test	0.97 (0.91-1.04)	0.03	.42	1.11 (1.01-1.22)	0.05	.04
Immunization	1.03 (0.98-1.08)	0.02	.28	1.01 (0.96-1.07)	0.03	.60

Abbreviations: GLM, generalized linear model; OOP, out of pocket; PCP, primary care physician.

^a^ Reference category was non–value-based insurance design preferred provider organization.

Among those with any utilization or positive allowed payments, the VBID PPO cohort had significantly higher mean total allowed payments per member per year for PCP office visits in both 2019 and 2020 (adjusted relative payments ratio, 1.05; 95% CI, 1.02-1.08; *P* < .001). The VBID PPO cohorts also had higher mean payments for laboratory test and medication OOP in 2020, but lower mean OOP payments for inpatient admission in 2020 (adjusted relative payments ratio, 0.90; 95% CI, 0.81-1.00; *P* = .05). The VBID and non-VBID PPO cohorts had no significant differences for the following utilization and payment measures in both 2019 and 2020: inpatient and outpatient combined, outpatient, prescription drug, OOP combined, emergency department, specialist, and psychiatrist visit.

We provide the estimated overall marginal effects in dollars from the fitted 2-part GLM models in eTable 2 in Supplement 1. The differential changes in per-member mean annual payments are small for most measures. Note that although the marginal effects are intuitively appealing in original measurement units, they are not unbiased DID estimates as the interaction item coefficients for GLMs.^15^

The VBID PPO subcohort, female and aged between 15 and 55 years old, had higher mean total inpatient cesarean-section positive payments than their non-VBID PPO counterpart in 2019 (Table 2). The sensitivity analysis for those with inpatient delivery yielded very similar results or conclusions (eTable 3 and 4 in Supplement 1).

The coefficients of interactions of the VBID PPO indicator and year indicator before 2019 in the 2-part DID GLMs are the model tests for parallel pre-VBID trends assumption for utilization and payments.^15^ They were insignificant for almost all measures after ATT weighting (eTable 5 in Supplement 1).

## Discussion

In this cohort study, we found that in 2019 or 2020, participation in the CalPERS VBID PPO was associated with (1) higher probability of PCP use, immunizations, and outpatient OOP payments, and lower probability of inpatient surgical and total admissions and OOP payments; (2) higher mean positive spending for PCPs, laboratory tests, inpatient cesarean deliveries and medication OOP payments; and lower inpatient OOP payments; and (3) similar total health care spending changes in both years, suggesting the VBID program has the potential to promote valued health care services while controlling costs for CalPERS PPO plan members.

The VBID association with some measures, such as PCP visits, immunizations, and inpatient surgical admissions, are as expected, while others are more nuanced. For example, the VBID PPO cohort experienced a higher chance of paying for outpatient visits OOP, but they also had lower positive outpatient OOP allowed payments (Table 2) and lower overall annual outpatient OOP allowed payments, although most are not significant (eTable 2 in Supplement 1). The VBID PPO cohort experienced a lower chance of paying for inpatient admissions OOP, lower positive inpatient OOP payments (Table 2), and lower overall annual inpatient OOP payments (eTable 2 in Supplement 1). These results suggest that even though the VBID PPO may be associated with a higher chance of outpatient OOP payments, it may come with lower annual OOP payments for both outpatient and inpatient services (Table 2; eTable 2 in Supplement 1).

Another unexpected result concerns cesarean sections. The low uptake rate for the healthy mother’s program makes drawing meaningful conclusions difficult regarding the VBID PPO higher positive mean total allowed payments for inpatient cesarean deliveries in a single year. Although statistically insignificant, the VBID PPO had a lower probability of inpatient cesarean deliveries than the non-VBID PPO (Table 2; eTable 3 in Supplement 1). Additionally, there were no essential differences in marginal effects on spending (eTable 2 and 4 in Supplement 1). It should be noted that the healthy mother’s program has no explicit goal of reducing cesarean delivery. Similarly, the VBID program’s significant association with positive laboratory test payments and medication OOP payments in 2020 may be due to the COVID-19 pandemic, more primary care interventions, or some other cause. Additional years of observation and study are warranted to further explore and confirm the real long-term VBID association with these measures.

The CalPERS VBID program needs improvement in scope and depth. One area is to enhance its member engagement. The high uptake rates in disease management participation and second opinion for surgery were primarily because VBID PPO members would automatically receive these incentives until they actively declined them. A similar approach may be used to address the low PCP uptake rate. For example, a PCP would be matched to a member if they do not choose a PCP upon open enrollment, but they still have the freedom to change or drop their assigned PCP anytime over the course of the year.

Improving VBID PPO member engagement with PCPs and participation in the incentivized activities should help achieve the program’s objectives. CalPERS could further increase the VBID PPO copayment differential between PCP and specialist visits by eliminating the copayment for assigned PCP office visits, thereby incentivizing VBID PPO members to select and use PCPs, promoting primary care and reducing expensive specialty and emergent care.^16,17,18,19^ Similarly, the VBID PPO may also automatically enroll all expectant mothers who do not explicitly decline program participation.

Another area for improvement is to target prescription drugs for high prevalence diseases, chronic conditions, or high-risk patients (eg, heart diseases, diabetes, hypertension, hyperlipidemia, and so on).^10,20,21,22,23^ Many VBID studies have found that lowering or eliminating cost sharing for prescription drugs would increase medication use, adherence, and spending for targeted diseases or conditions, although with uncertainty in total health care savings.^6,9,11,19,24,25,26,27,28,29,30,31,32,33,34,35,36,37^ CalPERS could consider combining reference pricing for prescription drugs with VBID to control total medication spending. For example, eliminating drug copayments if members choose the referenced prescription drugs for targeted diseases or conditions to lower member medication OOP payments and improve medication adherence. These measures will increase the operational complexity of administering pharmacy benefits and may face resistance from health plans and pharmacy benefit managers, which could be addressed and resolved during contract solicitation, negotiation, and renewal phases.

Increasing cost sharing for low-value care to reduce health care waste and cost may be another option for CalPERS to expand its VBID scope but with many challenges.^13,38,39^ Most VBID applications and studies have focused on decreasing cost sharing for high-value care and few have looked at increasing cost sharing for low-value care although the latter has been advocated since VBID’s inception.^40,41,42^ There are professional, ethical, and potentially legal issues regarding who and how to define, measure, and generalize usually situational health care as low value care, which may also increase administrative, operational, and benefit complexity, as well as member confusion.^43,44,45,46,47^ What’s more, its association with reducing total cost may not come to fruition in the short term. One empirical study found that substantially increased cost sharing for low-value services was associated with reduced targeted use but not with total cost savings.^48^ The literature suggests that clinician-based interventions are more effective than consumer-based interventions, and multicomponent interventions in addressing both clinician and patient roles have the greatest potential for reducing low-value care.^13,39^

### Limitations

Our VBID study focused on the cost and utilization of general health care providers and services in a large health benefit public purchaser’s commercial PPO health plans using rigorous methods and recent data, but with limitations, nevertheless. We applied a fixed cohort approach by requiring continuous enrollment in the VBID PPO or non-VBID PPO from 2017 through 2020 to reduce favorable selection bias due to lower VBID PPO premium and VBID incentives attracting healthy enrollees in 2019 and 2020.^15^ However, we may not exclude selection bias on unobserved factors without randomization even though we applied a DID ATT weighted regression adjustment. Therefore, this study cannot tell whether VBID associations with lower inpatient admissions and surgical procedures were from reduced unnecessary procedures or from limited access, although the former was more likely than the latter for these PPOs. Our study findings may not be generalizable to other states’ commercial health plans and the Medicare or Medicaid populations. The study time frame is short, and the COVID-19 pandemic had a shock on the 2020 results, which led to a lack of consistent patterns for many significant findings over the first 2 years. We also did not examine clinical outcomes, which warrant further research. The marginal effect sizes of most outcome measures are financially minimal. It may take a longer time and additional incentives targeting medications for certain conditions or populations for the CalPERS VBID PPO to materialize substantial cost savings. However, the VBID literature to date seem to imply that VBID is more effective in redistributing and enhancing health care value than reducing total cost.^6,28,49^

## Conclusions

In this study, the CalPERS VBID program was associated with desired changes for some interventions with no added total costs in its initial 2 years of operation: higher PCP and immunization utilization or spending, and lower inpatient total and surgical admissions and OOP payments. However, due to the small marginal effect sizes, the COVID-19 pandemic in 2020 and inconsistent results across years, additional years of observation and study are warranted to assure persistent VBID association. CalPERS may need to adjust current VBID interventions and keep exploring, implementing, and evaluating other VBID incentives and benefit designs to promote value while controlling cost for its health plan members.^50^

## Notes

Supplement 1. **eTable 1. **Comparison of VBID PPO and non-VBID PPO in 2018 and 2019 for Key Health Plan Characteristics **eTable 2. **Two-Part GLM Adjusted DID Marginal Effects (in Dollars) 2019-2020 **eTable 3. **Two-Part GLM Adjusted DID Estimates 2019-2020 for Any Inpatient Deliveries **eTable 4. **Two-Part GLM Adjusted DID Marginal Effects (in Dollars) 2019-2020 for Any Inpatient Deliveries **eTable 5. **Two-Part GLM Model Test Results for Parallel Pretrends Assumption 2017-2018

Supplement 2.
Data Sharing Statement
