# Supplementary material for: Association of Participation in a Value-Based Insurance Design Program With Health Care Spending and Utilization
Source: JAMA Netw Open. 2023 Mar 13;6(3):e232666. doi: 10.1001/jamanetworkopen.2023.2666 (PMC10011939; doi:10.1001/jamanetworkopen.2023.2666)
Supplement: Supplement 1. — eTable 1. Comparison of VBID PPO and non-VBID PPO in 2018 and 2019 for Key Health Plan Characteristics eTable 2. Two-Part GLM Adjusted DID Marginal Effects (in Dollars) 2019-2020 eTable 3. Two-Part GLM Adjusted DID Estimates 2019-2020 for Any Inpatient Deliveries eTable 4. Two-Part GLM Adjusted DID Marginal Effects (in Dollars) 2019-2020 for Any Inpatient Deliveries eTable 5. Two-Part GLM Model Test Results for Parallel Pretrends Assumption 2017-2018 [file jamanetwopen-e232666-s001.pdf]

Supplemental Online Content

Zhang H, Cowling DW. Association of participation in a value-based insurance design program with health care spending and utilization. *JAMA Netw Open*. 2023;6(3):e232666. doi:10.1001/jamanetworkopen.2023.2666

eTable 1. Comparison of VBID PPO and non-VBID PPO in 2018 and 2019 for Key Health Plan Characteristics

eTable 2. Two-Part GLM Adjusted DID Marginal Effects (in Dollars) 2019-2020

eTable 3. Two-Part GLM Adjusted DID Estimates 2019-2020 for Any Inpatient Deliveries

eTable 4. Two-Part GLM Adjusted DID Marginal Effects (in Dollars) 2019-2020 for Any Inpatient Deliveries

eTable 5. Two-Part GLM Model Test Results for Parallel Pretrends Assumption 2017-2018

This supplemental material has been provided by the authors to give readers additional information about their work.

© 2023 Zhang H et al. *JAMA Network Open.*

eTable 1. Comparison of VBID PPO and non-VBID PPO in 2018 and 2019 for Key Health Plan Characteristics

Benefit Design	VBID Plan		Non-VBID Plan	
	2018	2019	2018	2019
Premium, Single	$661	$492	$724	$765
Coinsurance	20%	20%	20%	20%
Deductible	$500	$1000	$500	$500
Maximum Individual Coinsurance (excluding pharmacy)	$3000	$3000	$3000	$3000
PCP Copay	$20	$10 with assigned PCP; $35 otherwise	$20	$20
Specialist Copay	$20	$35	$20	$35
Urgent Care Copay	$20	$35	$20	$35
Emergency Department Copay	$50	$50	$50	$50
Incentives to Lower Deductible	No	Yes, $100 per incentive	No	No

Notes: There were no changes in pharmacy benefits or copayments from 2018 to 2019 for both VBID and non-VBID plans. VBID: Value-based Insurance Design. PCP: primary care physician.

© 2023 Zhang H et al. *JAMA Network Open.*

eTable 2. Two-Part GLM Adjusted DID Marginal Effects (in Dollars) 2019-2020

	2019					2020				
**Total Allowed Payment**	**Marginal Effect**	**95% CI**		**SE**	**P**	**Marginal Effect**	**95% CI**		**SE**	**P**
Total Combined	-76.3	-511.4	358.9	222.0	0.73	352.4	-555.0	1259.8	463.0	0.45
Inpatient Admission	-218.2	-386.9	-49.5	86.1	0.01	-67.3	-283.9	149.3	110.5	0.54
Outpatient Visit	23.4	-169.4	216.1	98.3	0.81	-29.2	-305.0	246.5	140.7	0.84
Prescription Drug (Rx)	41.7	-23.2	106.6	33.1	0.21	46.3	-58.4	151.1	53.4	0.39
OOP Combined	-23.1	-51.4	5.3	14.5	0.11	-19.2	-53.9	15.6	17.7	0.28
Admission OOP	-8.8	-16.0	-1.6	3.7	0.02	-8.8	-15.6	-2.1	3.4	0.01
Visit OOP	-17.1	-42.3	8.2	12.9	0.19	-10.7	-41.9	20.4	15.9	0.50
Rx OOP	0.5	-1.5	2.5	1.0	0.61	1.9	-0.7	4.4	1.3	0.15
Inpatient										
Inpatient C-section	-0.1	-13.2	13.1	6.7	0.99	-5.5	-19.1	8.1	6.9	0.43
Inpatient Surgical	-121.7	-227.3	-16.1	53.9	0.02	6.7	-128.9	142.3	69.2	0.92
Outpatient										
PCP Office Visit	1.0	-3.8	5.8	2.5	0.69	10.7	5.6	15.8	2.6	0.000
Specialist Office Visit	-5.9	-11.9	0.1	3.1	0.05	-3.7	-10.9	3.6	3.7	0.32
Psychiatrist Outpatient	-0.4	-9.4	8.6	4.6	0.93	-5.0	-16.0	6.0	5.6	0.37
Emergency Department	27.8	-13.4	69.0	21.0	0.19	14.6	-23.9	53.2	19.7	0.46
Laboratory Test	-10.1	-19.9	-0.2	5.0	0.05	12.9	-2.0	27.9	7.6	0.09
Immunization	1.8	-0.1	3.6	0.9	0.06	1.0	-0.9	2.9	1.0	0.31

Notes: GLM: generalized linear model. DID: difference in differences. OOP: out of pocket. PCP: primary care physician. C-section: Cesarean section. CI: confidence interval. SE: standard error. Reference=Non-VBID PPO, 2018.

© 2023 Zhang H et al. *JAMA Network Open.*

eTable 3. Two-Part GLM Adjusted DID Estimates 2019-2020 for Any Inpatient Deliveries

**Logit GLM**	**2019**			**2020**		
**Total Allowed Payment**	**Relative Odds Ratio, 95% CI**	**SE**	**P**	**Relative Odds Ratio, 95% CI**	**SE**	**P**
Inpatient C-section	1.00 (0.52-1.93)	0.34	1.00	0.84 (0.44,1.62)	0.28	0.61
**Log-Gamma GLM**	**2019**			**2020**		
**Total Allowed Payment**	**Relative Payments Ratio, 95% CI**	**SE**	**P**	**Relative Payments Ratio, 95% CI**	**SE**	**P**
Inpatient C-section	1.30 (1.02-1.67)	0.17	0.04	1.00 (0.77,1.28)	0.13	0.98

Notes: Reference=Non-VBID PPO, 2018. GLM: generalized linear model. DID: difference in differences. C-section: Cesarean section. CI: confidence interval. SE: standard error.

eTable 4. Two-Part GLM Adjusted DID Marginal Effects (in Dollars) 2019-2020 for Any Inpatient Deliveries

	**2019**					**2020**				
**Outcome Measures**	**Marginal Effect**	**95% CI**		**SE**	**P**	**Marginal Effect**	**95% CI**		**SE**	**P**
Inpatient C-section	1729.1	-1725.0	5183.2	1762.3	0.33	-640.1	-4016.9	2736.8	1722.9	0.71

Notes: Reference=Non-VBID PPO, 2018. GLM: generalized linear model. DID: difference in differences. C-section: Cesarean section. CI: confidence interval. SE: standard error.

© 2023 Zhang H et al. *JAMA Network Open.*

eTable 5. Two-Part GLM Model Test Results for Parallel Pretrends Assumption 2017-2018

**Logit GLM**	**2017**			
**Total Allowed Payment**	**Relative Odds Ratio**	**95% CI**	**SE**	**P**
Total Combined	1.02	(0.96-1.08)	0.03	0.46
Inpatient Admission	0.96	(0.84-1.11)	0.07	0.62
Outpatient Visit	1.01	(0.96-1.07)	0.03	0.72
Prescription Drug (Rx)	1.03	(0.99-1.07)	0.02	0.19
OOP Combined	1.02	(0.97-1.08)	0.03	0.36
Admission OOP	0.95	(0.82-1.10)	0.07	0.48
Visit OOP	1.02	(0.97-1.07)	0.03	0.50
Rx OOP	1.01	(0.97-1.06)	0.02	0.50
Inpatient				
Inpatient C-section	0.79	(0.49-1.29)	0.2	0.35
Inpatient Surgical	0.93	(0.74-1.17)	0.11	0.55
Outpatient				
PCP Office Visit	1.04	(0.99-1.08)	0.02	0.09
Specialist Office Visit	0.95	(0.91-0.99)	0.02	0.03
Psychiatrist Outpatient	0.98	(0.92-1.04)	0.03	0.43
Emergency Department	1.06	(0.99-1.14)	0.04	0.10
Laboratory Test	1.02	(0.97-1.06)	0.02	0.43
Immunization	1.04	(0.99-1.09)	0.03	0.13
**Log-Gamma GLM**	**2017**			
**Total Allowed Payment**	**Relative Payments Ratio**	**95% CI**	**SE**	**P**
Total Combined	0.98	(0.93-1.02)	0.02	0.33
Inpatient Admission	1.04	(0.87-1.23)	0.09	0.67
Outpatient Visit	1.01	(0.96-1.06)	0.03	0.79
Prescription Drug (Rx)	0.94	(0.88-1.01)	0.03	0.07
OOP Combined	1.01	(0.97-1.05)	0.02	0.72
Admission OOP	1.01	(0.93-1.11)	0.05	0.75
Visit OOP	1.02	(0.98-1.06)	0.02	0.43
Rx OOP	0.99	(0.96-1.02)	0.02	0.44

© 2023 Zhang H et al. *JAMA Network Open.*

Inpatient				
Inpatient C-section	1.23	(0.99-1.52)	0.13	0.06
Inpatient Surgical	1.04	(0.76-1.41)	0.16	0.81
Outpatient				
PCP Office Visit	0.99	(0.97-1.01)	0.01	0.49
Specialist Office Visit	0.99	(0.96-1.01)	0.01	0.31
Psychiatrist Outpatient	0.95	(0.87-1.04)	0.04	0.29
mergency Department	1.01	(0.93-1.09)	0.04	0.84
Laboratory Test	1.05	(0.97-1.14)	0.04	0.19
Immunization	1.10	(1.05-1.16)	0.03	0.000

Notes: Reference=Non-VBID PPO, 2018. GLM: generalized linear model. DID: difference in differences. OOP: out of pocket. PCP: primary care physician. C-section: Cesarean section. CI: confidence interval. SE: standard error.

© 2023 Zhang H et al. *JAMA Network Open.*
