# Supplementary material for: Association of Participation in a Value-Based Insurance Design Program With Health Care Spending and Utilization
Source: JAMA Netw Open. 2023 Mar 13;6(3):e232666. doi: 10.1001/jamanetworkopen.2023.2666 (PMC10011939; doi:10.1001/jamanetworkopen.2023.2666)
Supplement: Supplement 2. — Data Sharing Statement [file jamanetwopen-e232666-s002.pdf]

Data Sharing Statement

Zhang. Association of Participation in a Value-Based Insurance Design Program With Health Care Spending and Utilization. *JAMA Netw Open*. Published March 13, 2023. doi:10.1001/jamanetworkopen.2023.2666

Data
Data available: No
